# Tight bag

**DOI:** 10.4103/0019-5049.65354

**Published:** 2010

**Authors:** S Parthasarathy, M Ravishankar

**Affiliations:** Government District Headquarters Hospital, Kumbakonam - 612 001, Tamil Nadu, India; 1 Department of Anaesthesiology, Mahatma Gandhi Medical College and Research Institute, Pondicherry, India

**Keywords:** Anaesthesia, breathing system, reservoir bag, tight bag

## Abstract

Tight bag is a clinical situation where excessive pressure needs to be applied to a reservoir bag of a breathing system to an intubated patient, which may or may not produce satisfactory ventilation. The various clinical scenarios and the appropriate steps for its prevention are described.

## INTRODUCTION

Tight bag is a clinical situation where excessive pressure needs to be applied to a reservoir bag of a breathing system to an intubated patient, which may or may not produce satisfactory ventilation. In these days of anaesthesia ventilators, the feel of hand of reservoir bags and tight bags are decreasing among younger generations of anaesthesiologists. This condition may be defined as an unacceptable increase in airway pressure to maintain the satisfactory tidal volume. Usually, the airway pressure is around 15-20 cm to deliver a volume of 8 mL/kg. But if it crosses 25 cm, the clinical scenario of tight bag will arise. The clinical situation of a tight bag can occur in three settings in anaesthesia.

- Immediately after intubation
- During maintenance of anaesthesia
- During extubation.

The causes of tight bag can be grouped into two categories:

- Anticipated
- Unanticipated

The condition, especially if it occurs unanticipated, that too if the anaesthesiologist is unable to maintain satisfactory ventilation, he or she has to miss a heart or two with an additional burden of potential and imminent life risk to the patient. It is ideal to have protocols to manage such emergencies.

## CAUSES OF TIGHT BAGS IMMEDIATELY AFTER INTUBATION

**Mechanical**

- Bag twisting at neck
- High flows, O_2_ flush
- Obstruction in tube
- Obstruction in circuits
- Smaller tube
- Malfunctioning valves
- Foreign bodies, e.g., tooth, adenoids

**Pathologic**

- Hyperreactive airways
- Bronchial asthma
- Anaphylaxis
- COPD
- Aspiration
- Ascitis
- Endobronchial intubation, light anaesthesia
- Extrinsic compression from tumors
- Pleural effusion
- Obesity, smoking

### During maintenance of anaesthesia

#### Anticipated causes

- Recovery from relaxants
- Endotracheal tube in carina
- Pulmonary edema in cardiac patients
- Acute mitral regurgitation
- Light anaesthesia
- Bronchospasm in asthmatics
- Drug injection, e.g., prostadin, blood, non steroidal anti inflammatory drugs (NSAIDs)

#### Unanticipated causes

- Amniotic embolism
- Fat embolism
- Pneumothorax
- Acid aspiration
- Anaphylaxis
- Mechanical

### During recovery from anaesthesia

- Breath holding
- Bucking
- Bronchospasm - possible neostigmine effect
- Interstitial edema due to improper intravenous fluids
- Biting the tube, this may be significant in pediatrics

The various causes may be basically divided as mechanical and pathologic causes for the purpose of understanding the pathophysiology and their prevention.

### Mechanical causes

The mechanical causes are usually unanticipated. Twisting of the bag at the neck is one of the commonest causes of tight bag. This is usual when the bag has a long neck. A little rotation of the bag while squeezing can untwist and release the tight bag to ease ventilation. High flow rates can occur if we keep high flows to maintain satisfactory ventilation with leaky masks prior to intubation and continue the flows after intubation. Oxygen flush may be activated to compensate for an ill fitting mask and failure to turn it off after intubation is another cause. In newer machines, it is only possible to keep oxygen flush either in on or off position but in older models the intermediate higher flows are possible followed by a tight bag.[1] Obstruction of the endotracheal tube, catheter mounts or breathing systems are common situations affecting ventilation.[2] A variety of foreign bodies are reported.[3,4] Broken ampoules are recovered from circuits. It is incorrect to use adaptors or metal connectors to cut ampoules. The use of snap off ampoules and the proper disposal of the remains of ampoules should be insisted instead of keeping them in Boyle’s machine, to avoid critical mishaps. With the transparent tubes and circuits, it is possible to decrease foreign bodies and problems. The connectors can still be a cause of problem. There is an endless list of foreign bodies reported in literature. During nasal intubation for surgeries like tonsillectomy, the procedure of intubation may dislodge an adenoid and this will enter into the endotracheal tube. An avulsed nasal turbinate, thick secretions or blood may cause partial obstruction to the tube. It is important to remember a dislodged tooth as a reason for the tight bag situation. In all of these situations, the anaesthesiologist may experience a tight bag scenario. It will mimic a bronchospasm and the patient may desaturate fast. Unless there is a quick intervention, we may land up in an anaesthetic disaster. Another important cause of tight bag is endobronchial intubation. Usually, the tube passes into the right bronchus crossing the exit of upper lobe branch to ventilate only two lobes. This can result in a higher resistance to produce a tight bag. An endobronchial intubation can be during a position change after intubation.[5] Hence, it is mandatory to check air entry immediately after intubation in both axillae, followed by a similar check after each position change. With the increasing number of laparoscopic surgeries and their associated position changes, the tight bag situation may arise frequently. Hence, it is essential to check for correct position of the tracheal tube. In such patients with positional changes, the need for using the mark in the tube to idealize the position during intubation itself becomes mandatory. A smaller size tube can be a cause problem sometimes. With a smaller tube, the resistance increases. As evident from Poisiuele’s law, the resistance may increase by a fourth power of radius of a tube.

Poisiuele’s law *R* = 8*nl /πr*^4^ (*n* - Viscosity, *l* - Length)

While using low-volume high-pressure cuffs, overinflation of the cuff might compress the endotracheal tube and reduce its lumen. A high-volume cuff, especially when weak, can herniate to the tip to cause obstruction. This is possible when a tube after inflation of the cuff is pulled to adjust the position. A soft endotracheal tube could kink in the intraoral position when the neck is flexed or it could kink at the level of incisors if the extraoral portion of the tube is large and there is a drag on the tube. A carinal intubation or the tube tip impinging on the wall of the trachea can cause tight bags. Using Murphy tubes rather than Magill tubes can avoid a few problems.[6]

Another tube causing difficulties is the double lumen tube.[7,8] Various malpositions are described in books and journals, but a few are not precisely described.

- Kinking of the endobronchial portion of the tube. This can occur if the tube is not positioned properly and the surgeons pull the opposite bronchus during dissection.
- Obstruction of the tracheal lumen by the tracheal wall itself. This can happen if a bigger size tube is forced inside and dilation of trachea happens. The bronchial portion will stay in the trachea and the tracheal wall will collapse on the tracheal lumen of the double lumen tube.

In these days of fiber optics, it is necessary to check the insertion of double lumen tubes with the help of fiber optic laryngoscope.

#### Malfunctioning of the valve

Any breathing system should vent the amount of gases into the atmosphere as it inflows. If the venting is less or the flows are more, a tight bag can occur. This is characteristic when the adjustable pressure limit (APL) valve gets stuck in the closed position. The spring of the Heidbrink valve can be a culprit sometimes. The condensation of the water vapor in a circle system can make a valve stick in the inspiratory position. This will result in total rebreathing and tight bag. While using a non-rebreathing circuit and valve, a wrongly connected valve or a malfunctioning valve may lead to a dangerous tight bag situation.[9]

In some occasions, the surgeon may be the culprit. During cleft lip, palate surgeries, the gag introduced by the surgeon may compress the tube to cause tight bag. During head and neck surgeries, after intubation, the towels are tightly wrapped around the tube to produce kinking. The use of nonkinkable tubes in appropriate cases may reduce the problem.[10]

During extubation, after the administration of reversal, the patient can bite the tube and it may cause havoc as tight bags and inability to extubate will follow. Hence, a smooth and uneventful intubation and extubation will solve and prevent major tight bag situations.

## PATHOLOGIC CAUSES

The etiology and the pathogenesis of pathologic causes are varied. Though they may occur during induction, maintenance or extubation, which is useful for immediate action and prevention, they may be described as the following groups for the purpose of discussion.

- Intrinsic airway narrowing
- Increased airway resistance and decreased compliance
- Extrinsic narrowing of airways.

### Intrinsic airway narrowing

#### Hyperreactive airways

It is to be mentioned clearly here that this is different from asthma. It was found that this condition can exist in 20% of blood relatives of asthmatics. The clinical condition can occur with a viral infection of the respiratory tract. Following a viral infection, the increase in airway responsiveness is found even in normal subjects. The hyperreactiveness can last for 2-8 weeks after a viral respiratory illness. Increased vagal induced bronchoconstriction and excessive response to tachykinins are the described possible reasons for a postviral hyperresponsiveness. An anaesthesiologist may encounter a patient with repeated viral infections. Though presently normal, such a patient after intubation can develop an inordinate high inflation pressure.[11]

There are two important conditions which manifest as hyperresponsiveness.

- Pulmonary aspiration of gastric contents
- Anaphylaxis to injected drugs.

#### Aspiration

This can happen during induction due to passive regurgitation of gastric contents as the oesophageal sphincters relax. It is more likely in certain situations.

Emergencies, pregnancy, intestinal obstruction and abdominal tumors are some of the clinical conditions. The initial event is a chemical reaction of the mucosa of the trachea and bronchi leading to mucosal edema and bronchospasm. If the pH is 2.5 and the volume is more than 25 mL, the reaction is extensive. Initially, the injury to the alveolocapillay membrane results in a leakage of liquids, proteins and cellular components into the interstitial space and progresses to alveolar space. This leads to decrease in lung compliance and the need to use higher inspiratory pressures to inflate the lungs. This may take a few minutes to few hours for the reaction to set in according to the severity of aspiration, but mucosal edema and neutrally mediated bronchoconstriction may be instantaneously evident. The neutrally mediated bronchoconstriction may happen even with nonacid liquids leading to a tight bag scenario. Prophylaxis against aspiration and measures to prevent its severity should be followed in all cases especially in individuals with a risk of aspiration. Early identification and management of this cause of tight bag should be done in all patients.[12]

#### Anaphylaxis

This could be of two types.

1. Minor anaphylactoid reaction with edema of airways. Usually frank bronchospasm is rare
2. Major reactions with hypotension and bronchospasm to injected drugs can occur to produce tight bags.

Minor histamine release with drugs like morphine and atracurium do occur but these are usually not very severe to produce tight bags. It is ideal to remember that anaphylaxes can also occur to colloids and cement of surgeons.[13,14]. Fisher and Baldo[15] after analysis of 460 cases of anaphylaxes during anaesthesia over a period of 20 years have published certain data which are given below:

1. Cardiovascular collapse is the most common finding usually presenting with hypotension and tachycardia.
2. Bronchospasm is present in 30% (166) of cases.
3. Out of the 166 cases, in 117, the first presenting feature was difficulty in inflation of the lungs.
4. In 13 of them tight bag was the only feature.

Hence, it is worthwhile to remember anaphylaxis to intravenous drugs as one of the causes of tight bag situations.

#### Bronchial asthma, COPD and smokers

All these conditions produce obstructive airway disease which may or may not be revealed in a preoperative examination.[16] Exposure especially prolonged to dust, smoke, allergens can cause release of mediators of intense inflammatory reaction and bronchoconstriction. Patients get sensitized over a period of time and later a trivial stimulus can precipitate an episode of severe increase in airway resistance to produce tight bags. It is ideal to optimize the wheezing patients with described protocols including bronchodilators like theophyllines, nebulized steroids and beta 2 agonists. It is better to take prophylactic measures to decrease airway reflexes during intubation. Certain drugs on like nonsteroidal anti inflammatory drugs, prostadin and neostigmine, can produce mild bronchospasm especially in prone patients to cause increased inspiratory pressures for adequate ventilation.[17–19]

#### Pulmonary embolism

Two distinct entities should be thought of as a cause of intrinsic airway narrowing during anaesthesia.

1. Amniotic fluid embolism[20]
2. Fat embolism.[21]

The lung vasculature acts as a filter of all the materials which gain entry into the venous system. Amniotic fluid can access into the circulation during cesarean section immediately after delivery of the baby. Fat can enter the circulation during orthopedic manipulations and both these conditions can present as difficulty in ventilation, hypotension and hypoxemia. Embolic obstruction produces an intrapulmonary dead space resulting in areas of no gas exchange. This is compensated by constriction of air spaces to counter ventilation perfusion mismatch. If the embolism is massive, there may be sudden death. But if it slow and diffuse, the presentation is one of bronchospasm along with hypoxemia and hypotension. Patients can present with tight bags as one of the main clinical features.

### Increased airway resistance with decreased compliance

#### Excessive thick secretions

This can occur in patients who had a recent respiratory illness or due to inhalation of vapors of ether. As ether is almost outmoded except in rare peripheral centers, this cause becomes rare. Sometimes repeated manipulation of trachea in unatropinized patients can give rise to tight bags with excessive secretions.[22]

#### Pulmonary edema

Irrespective of the cause, pulmonary edema causes reduction of compliance; when severe, fluid comes to alveoli to cause increased airway resistance. In susceptible patients, e.g., with mitral valvular disease or postinfarction status,[23] the cause of a sudden tight bag can be construed as pulmonary edema and managed in the same way. Anaesthetics can interact with drugs including antipsychotics and produce pulmonary edema.[24] An acute mitral regurgitation can occur in patients with closed mitral valvotomy[25] immediately after the procedure. Prompt recognition of complication is a must to manage these cases.

### Extrinsic narrowing of airways

Intubation in light anaesthesia, bucking on the tube, recovery from relaxants either during maintenance or recovery can produce reflex airway narrowing due to increase in intrapleural pressure/intrathoracic pressure. This is brought about by sustained expiratory muscle contraction and reduced chest wall compliance. Clinical conditions like obesity, massive ascitis, pregnancy and massive abdominal tumors[26] can compress the lung parenchyma and airways by increased intra-abdominal pressure and diaphragmatic splinting. A latest addition to this is creation of voluntary pneumoperitoneum in cases of laparoscopic surgeries.[27] This is countered by gasless abdominal lift laparoscopic surgeries. The tight bag situation will clear as soon the condition of excess intra-abdominal pressure goes off.

The other conditions which can produce compression of lungs from outside are pleural effusion and pneumothorax.[28,29] The effusion can be diagnosed in the preoperative period and the tight bag is usually anticipated. In case of pneumothorax, it can come unanticipated. It can be due to a rupture of an emphysematous bulla or barotraumas with high flows due to equipment malfunction. Lung injury can be a part of unsuccessful multiple central venous cannulation or brachial plexus block.[30] This can manifest after institution of positive pressure ventilation as tight bags. High level of caution should be exercised while anaesthetizing thoracic injuries and neonates with hypoplastic lungs[31] as pneumothorax and tight bags may present and the condition may worsen rapidly if not for urgent remedial measures.

### Laryngeal mask and high inflation pressures

The laryngeal mask airway (LMA), a device designed for upper airway management, serves as a bridge between a face mask and an endotracheal tube.[32,33] So far, the LMA has been used in millions of patients and is accepted as a safe technique in a large variety of surgical procedures. Hence, it is necessary to mention a few specific clinical situations peculiar to using LMA. Even though it is advantageous and it has become universal, there are a few described complications. During insertion of LMA, laryngospasm and coughing may result from inadequate anaesthesia, tip impaction against the glottis or aspiration. Inability to ventilate the lungs may also result from inadequate anaesthesia, or inappropriate mask size, and high airway pressures. These situations may present with tight bags even when the clinical airway is appropriate with LMA, leave alone malposition and airway obstruction. Displacement of the LMA after insertion may be caused by inadequate anaesthetic depth, a pulled or twisted tube, and inadequate mask size. The above mentioned causes along with trivial and slow aspiration may cause tight bags during maintenance. Problems during emergence include laryngospasm and coughing when oral secretions enter the larynx following cuff deflation or removal of the LMA at an inappropriate anaesthetic depth, tube occlusion by biting, and regurgitation. Effects of pharyngolaryngeal reflexes such as laryngospasm, coughing, gagging, bronchospasm, breath holding and retching may also be associated with usage of an LMA. All these can present with tight bag scenarios with the use of normally positioned LMAs. Selection of appropriate size, introduction at adequate depth, developing necessary skills of insertion and removal are some of the answers to the above problems.

To conclude:

1. Tight bags especially with unsatisfactory ventilation may cause a nightmare to anaesthesiologists.
2. It is ideal to form protocols and approach the problem in a systematic manner.
3. There is no alternative to sticking to basics which includes a thorough machine and circuit check before anaesthetizing all cases.
4. It is better to keep Ambu bags and alternate breathing systems.
5. It is mandatory to optimize the medical condition before any elective case.

We have to admit that this article is an attempt to give an overall review of a clinical scenario with possible incidences and preventive measures. The exhaustive details of each instance are not covered. The highlight is on listing, prevention and treatment of causes with a possible management protocol to enlighten postgraduates and practising anaesthesiologists who come across such instances to prevent them from landing up in mishaps. The list of causes may be endless and continued reporting of such instances will go on.
